# Extended Reality for Image-Guided Small Animal Surgery: Current Evidence, Clinical Applications, and Future Directions

**DOI:** 10.3390/ani16193120

**Published:** 2026-10-05

**Authors:** Domenico Caivano, Federica Valeri, Andrea Corda, Antonello Bufalari

**Affiliations:** 1 Department of Veterinary Medicine, University of Perugia, Via San Costanzo 4, 06126 Perugia, Italy; federica.valeri@dottorandi.unipg.it (F.V.); antonello.bufalari@unipg.it (A.B.); 2 Department of Veterinary Medicine, University of Sassari, Via Vienna 2, 07100 Sassari, Italy

**Keywords:** virtual reality, augmented reality, mixed reality, canine, sectional imaging, veterinary surgery

## Abstract

Image-guided surgery has become an integral component of modern veterinary surgery using advanced imaging modalities, such as computed tomography, magnetic resonance imaging, ultrasonography, and fluoroscopy. Recently, extended reality (XR) technologies have emerged as promising tools to enhance image-guided surgical procedures by improving three-dimensional anatomical understanding, surgical navigation, and intraoperative decision-making. Although XR has gained considerable attention in human surgery, its application in veterinary surgery remains at an early stage. This narrative review aimed to provide a comprehensive overview of the current evidence regarding XR technologies in small animal surgery, with particular emphasis on their integration with preoperative and intraoperative imaging modalities. The available literature was reviewed to evaluate current clinical applications, technological developments and future perspectives. A literature search was performed in PubMed, Scopus, and Web of Science for studies published up to May 2026 using combinations of terms related to extended reality and small animal veterinary surgery. Original articles, case reports, pilot studies, technical reports, and reviews addressing clinical, experimental, or simulation-based applications were considered. Although several studies have explored the use of XR technologies for veterinary education, anatomical training, and diagnostic imaging instruction, relatively few investigations have assessed their direct application in clinical surgical practice. Potential applications have been described in orthopedic, oncologic, neurologic, cardiovascular, and minimally invasive procedures. Crucially, the currently available peer-reviewed evidence is restricted to canine patients, as no eligible feline studies were identified. Current evidence suggests that XR is an emerging technology with potential applications in canine surgical planning, image-guided procedures, and surgical training. Further prospective clinical studies are required to establish its clinical effectiveness, cost-effectiveness, and impact on surgical outcomes.

## Simple Summary

Modern veterinary surgery increasingly relies on advanced imaging techniques to better understand patient anatomy and improve surgical planning. Extended reality (XR) offers innovative approaches by allowing to visualize three-dimensional anatomical structures and interact with patient-specific models. This review summarizes the current use of XR technologies in small animal surgery and evaluates their potential benefits and limitations. Although XR has shown promising results in veterinary education, anatomical training, and imaging interpretation, its direct application during clinical surgical procedures remains limited. While current evidence suggests that XR could support more precise and personalized surgical approaches in the future, further studies are needed to confirm its accuracy, practical feasibility, and clinical impact.

## 1. Introduction

Image-guided surgery relies on the integration of imaging data into the surgical workflow, allowing clinicians to visualize anatomical structures and pathological lesions before and during a surgical procedure [1,2,3]. Advanced imaging modalities have progressively expanded the ability of veterinary surgeons to perform minimally invasive, interventional and anatomically complex procedures. In small animal veterinary medicine, computed tomography (CT), magnetic resonance imaging (MRI), ultrasonography (US), fluoroscopy, and CT angiography provide complementary information for anatomical characterization, lesion localization, procedural planning, and intraoperative guidance. Ultrasound, for instance, can provide real-time guidance during interventional procedures such as transhepatic vascular embolization [4] or localization and surgical management of vegetal foreign bodies in dogs and cats [5,6]; multimodal imaging can support the surgical management of foreign bodies in anatomically complex regions [7]. Additionally, ultrasound-guided interventions have expanded into interventional oncology; for instance, thermal high-intensity focused ultrasound has been successfully utilized for the non-invasive ablation of solid tumors in canine patients [8] and soft tissue sarcomas in cats [9]. Fluoroscopy and other imaging-based techniques have also contributed to the development of image-guided and minimally invasive interventions in dogs and cats [3,10,11]. Advanced image-guided techniques can therefore improve lesion localization, anatomical assessment and procedural planning, particularly when direct surgical visualization provides limited information in canine and feline patients [1,2,3,4,5,6,7,8,9,10,11,12].

Despite these advances, surgeons are still often required to mentally translate two-dimensional imaging information into a three-dimensional (3D) understanding of complex anatomical relationships. Recent developments in digital visualization technologies have led to the emergence of extended reality (XR), an umbrella term encompassing virtual reality (VR), augmented reality (AR), and mixed reality (MR). These technologies aim to bridge the gap between medical imaging and surgical execution by providing immersive, interactive, and spatially intuitive visualization of patient-specific anatomy [13,14]. VR creates a fully digital environment in which users can interact with 3D anatomical models derived from imaging datasets. AR overlays virtual information onto the real-world environment, enabling surgeons to visualize relevant anatomical structures while maintaining direct interaction with the patient. MR further expands these capabilities by allowing virtual objects to be anchored within and interact dynamically with the physical environment [13,14,15]. Collectively, these technologies provide different levels of interaction between virtual content and the physical environment, making them suitable for different stages of the surgical workflow.

In human medicine, XR technologies have experienced rapid growth over the past decade and have been investigated in numerous surgical disciplines, including neurosurgery, orthopedic surgery, cardiovascular interventions, maxillofacial surgery, hepatobiliary surgery, and surgical oncology [13,15,16]. Reported benefits can include improved anatomical understanding, enhanced surgical planning, reduced operative times, increased procedural accuracy, and improved educational opportunities for trainees [13,14,15]. Furthermore, the combination of XR with surgical navigation systems, artificial intelligence, robotic platforms, and advanced image-processing algorithms is increasingly being investigated as a component of precision surgery [14,15,16,17,18,19].

In contrast, the application of XR technologies in veterinary medicine remains limited. Although recent reviews have summarized the use of XR technologies in veterinary medicine, they have primarily focused on educational applications and anatomical teaching rather than on their integration within image-guided surgical workflows [20,21].

Therefore, the purpose of this review is to provide a comprehensive overview of the current applications of XR technologies in image-guided small animal surgery. Emphasis is placed on the integration of XR with contemporary imaging modalities, its potential role in surgical planning and intraoperative guidance, current limitations hindering widespread adoption, and future developments. By critically examining the available evidence, this review aims to highlight both the opportunities and challenges associated with the implementation of XR technologies in veterinary surgical practice.

## 2. Materials and Methods

This narrative review was conducted following the general methodological principles proposed for narrative literature reviews [22]. A comprehensive search of the veterinary literature was performed using the PubMed, Scopus, and Web of Science databases up to May 2026 to identify publications addressing the application of XR technologies in veterinary surgery.

The following keywords and combinations of terms were used: (“extended reality” OR “virtual reality” OR “augmented reality” OR “mixed reality”) AND (“veterinary” OR “canine” OR “dog” OR “feline” OR “cat”) AND (“surgery” OR “surgical” OR “image-guided surgery” OR “navigation” OR “intervention”). Original articles, case reports, pilot studies, technical reports, and review articles published in English were considered. Priority was given to studies describing the clinical application of XR technologies in small animal surgery, including preoperative planning, intraoperative navigation, and image-guided interventions.

Given the limited number of clinically oriented studies currently available, publications describing XR-based surgical simulation and procedural training were also considered when they provided insights into technologies directly applicable to image-guided surgical workflows. Studies exclusively focused on general veterinary education, anatomy teaching, or non-surgical applications were considered only when they contributed to understanding the current development and potential clinical translation of XR technologies.

Because this study was designed as a narrative review, no formal systematic screening protocol, meta-analysis, or standardized risk-of-bias assessment was performed. To ensure a transparent selection workflow, all retrieved records were initially screened by title and abstract by two authors (D.C. and F.V.), followed by a comprehensive full-text assessment of eligible articles to determine final inclusion. The literature was qualitatively appraised according to study design, clinical versus experimental setting, XR technology, imaging modality, surgical application, and reported outcomes. Studies involving human patients or large animals were not considered part of the clinical evidence synthesis.

## 3. Results

The literature search identified a growing body of publications addressing XR technologies in veterinary medicine. Most of the available literature focused on educational applications, anatomical visualization, simulation-based training, and technical development of immersive visualization systems. In contrast, relatively few studies described the direct clinical application of XR in small animal surgery. Moreover, the search strategy was designed to identify studies investigating XR applications in small animal surgery, including both canine and feline species. Although both species were included in the search strategy, the relevant literature identified in the present review consisted exclusively of canine studies; no relevant feline studies were identified.

**Figure 1:**
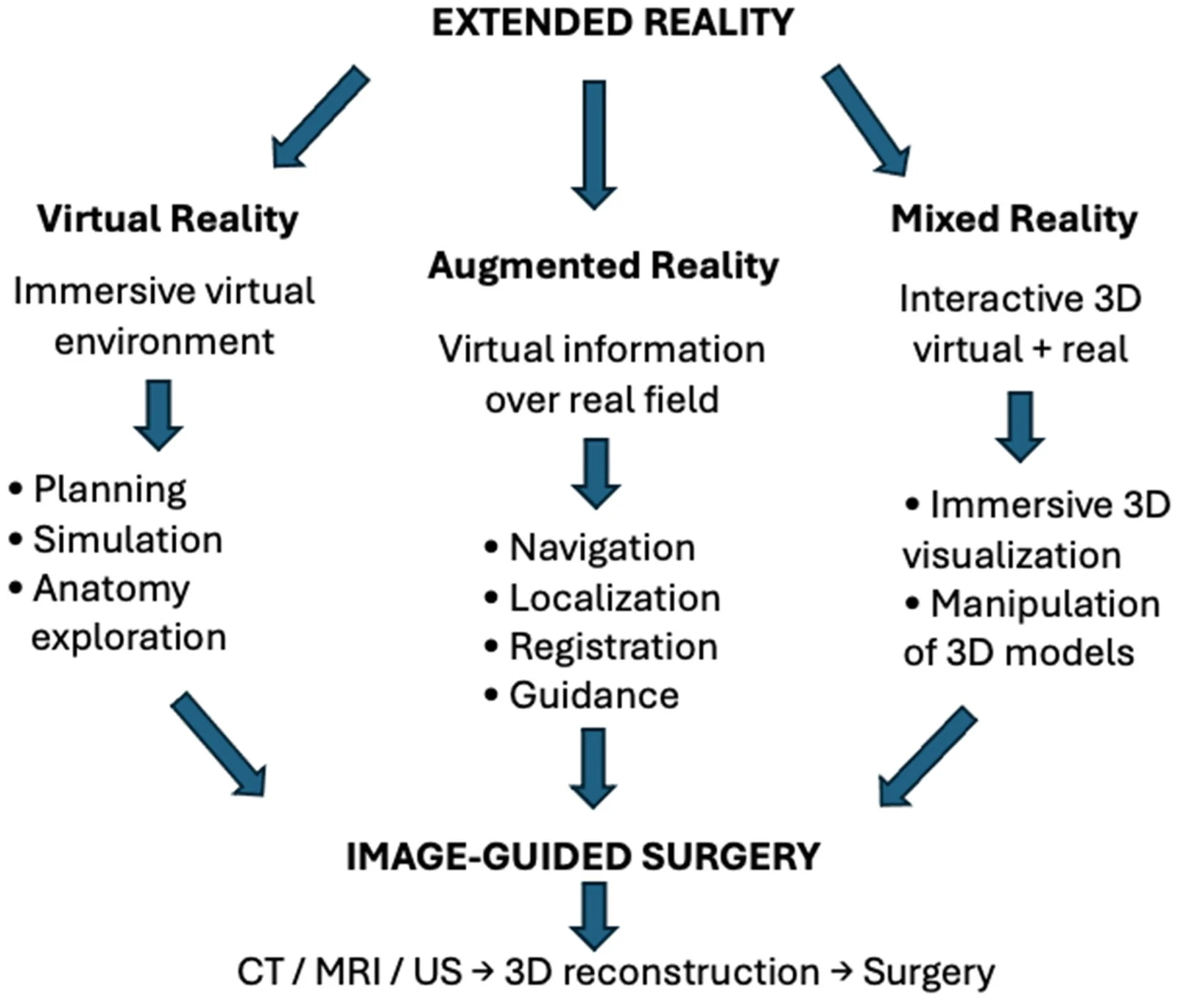
Extended reality technologies in image-guided veterinary surgery. Schematic representation of virtual reality, augmented reality, and mixed reality and their main applications in surgical planning and intraoperative guidance. CT, computed tomography; MRI, magnetic resonance imaging, US, ultrasound; 3D, three-dimensional.

The publications considered in this review encompassed original research, technical reports, pilot studies, case reports, and reviews. Clinical applications were primarily related to preoperative surgical planning, intraoperative navigation, image-guided interventional procedures, and selected neurosurgical, cardiovascular, orthopedic, and oncologic applications. VR, AR, and MR represent distinct but partially overlapping approaches within the XR spectrum, with different potential roles across the surgical workflow (Figure 1).

For the purposes of this review, XR was used as a term encompassing VR, augmented reality AR, and MR systems that provide interactive or immersive visualization and/or spatial integration of virtual information with the physical environment. Conventional two-dimensional imaging, standard non-interactive volume rendering, and three-dimensional reconstructions without an XR interface were not considered XR technologies *per se*. Interactive stereoscopic three-dimensional platforms were considered XR-related immersive visualization when they provided interactive spatial visualization but did not meet the defining characteristics of VR, AR, or MR. Systems such as EchoPixel True 3D were classified according to the specific configuration and mode of interaction described in the original study.

Given the heterogeneity of the available veterinary literature in terms of study design, technologies employed, and clinical objectives, a qualitative narrative synthesis was considered the most appropriate approach for critically discussing the available evidence. Educational studies directly related to surgical simulation, image-guided procedures, or operative workflow were included to illustrate the current maturity of XR technologies and their potential role in facilitating future clinical translation.

Studies describing direct clinical applications are summarized in Table 1, whereas educational and simulation-based applications are discussed separately, as they represent an important developmental step toward the future implementation of XR-assisted surgery.

The included studies were considered within three main categories: clinical canine studies, experimental canine studies, and simulation or educational studies. Clinical and experimental studies are discussed according to their principal surgical application, whereas simulation and educational studies are discussed separately.

### 3.1. Current Applications of Extended Reality in Canine Surgery

#### 3.1.1. Extended Reality for Preoperative Surgical Planning

Preoperative planning currently represents the most advanced and clinically developed application of XR technologies in canine surgery. The conversion of CT and MRI datasets into interactive 3D environments enables surgeons to visualize patient-specific anatomy in a more intuitive and immersive manner than conventional multiplanar imaging. Rather than replacing standard imaging modalities, XR complements existing diagnostic workflows by improving spatial perception, facilitating multidisciplinary communication, and enhancing surgical strategy development before entering the operating room [20,21,23].

Cardiovascular surgery represents one of the earliest areas in which immersive visualization demonstrated potential clinical value in veterinary medicine. Stoner et al. reported an early application of stereoscopic 3D visualization for evaluating atrial septal defects in five dogs. The interactive visualization platform enabled clinicians to manipulate patient-specific cardiac reconstructions in real time, substantially improving the perception of intracardiac anatomy compared with conventional two-dimensional echocardiographic images. Although the system was primarily designed as a diagnostic and planning tool rather than an intraoperative navigation platform, the study highlighted the potential of immersive visualization to facilitate therapeutic decision-making in complex congenital cardiovascular disease [24].

Saunders et al. demonstrated the potential of CT angiography-derived immersive 3D visualization for the evaluation of canine patent ductus arteriosus morphology. Using EchoPixel True 3D together with conventional volume-rendering software, the authors generated interactive patient-specific reconstructions that allowed free manipulation of cardiovascular anatomy, facilitating comparison with conventional angiographic and echocardiographic views. Although the technology was not used for real-time intraoperative navigation, the study highlighted the potential value of immersive XR visualization for procedural planning, device selection, and preoperative understanding of complex cardiovascular anatomy, representing an early example of XR-assisted planning in veterinary interventional cardiology [25].

**Table 1:** Summary of original studies investigating extended reality (XR) technologies for image-guided canine surgery, detailing authors, sample characteristics, XR modalities, imaging modalities, surgical applications, and main findings.

Authors (Year)	Sample	XR Technology	Imaging/Data Source	Surgical Application	Main Findings
Stoner et al. (2025) [24]	5 dogs	Interactive stereoscopic 3D visualization	ECG-gated CT angiography	Assessment and planning of ASD intervention	Interactive 3D visualization facilitated detailed assessment of ASD morphology and spatial relationships and supported procedural planning
Cupido et al. (2025) [26]	1 dog	VR + AR	CT and echocardiography	Surgical correction of complex vascular anomaly	VR facilitated preoperative planning, while AR provided intraoperative anatomical guidance, supporting successful surgical correction
Tipirneni et al. (2026) [27]	Experimental model	AR glasses	3D canine head model	Spatial tracking and surface annotation	AR improved real-time spatial tracking and enabled direct surface-level annotations, supporting potential applications in surgical navigation
Zhou et al. (2017) [28]	Experimental model	AR-assisted robotic surgery	Preoperative imaging 3D reconstruction	Mandibular angle split osteotomy	AR-assisted robotic guidance enabled accurate localization and execution of predefined osteotomy planes, demonstrating the feasibility of AR-guided robotic surgery
Li et al. (2021) [29]	Experimental model	AR navigation	Patient-specific CT	Pulmonary nodule localization before thoracoscopic surgery	Patient-specific 3D reconstructions were registered with the surgical field, allowing accurate localization of pulmonary targets and reducing localization errors
George et al. (2023) [30]	Experimental model	MR display	Fluorescence and multispectral imaging	Fluorescence-guided surgery	Real-time fluorescence information was displayed as MR overlays, enabling spatial visualization of surgical targets and demonstrating feasibility for intraoperative guidance

XR, extended reality; AR, augmented reality; VR, virtual reality; MR, mixed reality; CT, computed tomography; ASD, atrial septal defect; 3D, three-dimensional.

The first clinical implementation of a complete XR workflow in small animal surgery was later reported by Cupido et al., who described the management of a dog presenting with a complex congenital vascular anomaly. Following CT angiography, patient-specific vascular structures were segmented to generate an immersive 3D model that could be explored interactively before surgery. The XR environment enabled surgeons to appreciate the complex anatomical relationships between the anomalous vessels and surrounding structures, facilitating surgical planning and improving intraoperative orientation. Although limited to a single case report, this study demonstrated the clinical feasibility of integrating XR into the surgical management of canine patients and represented an important proof-of-concept for future clinical applications [26].

**Figure 2:**
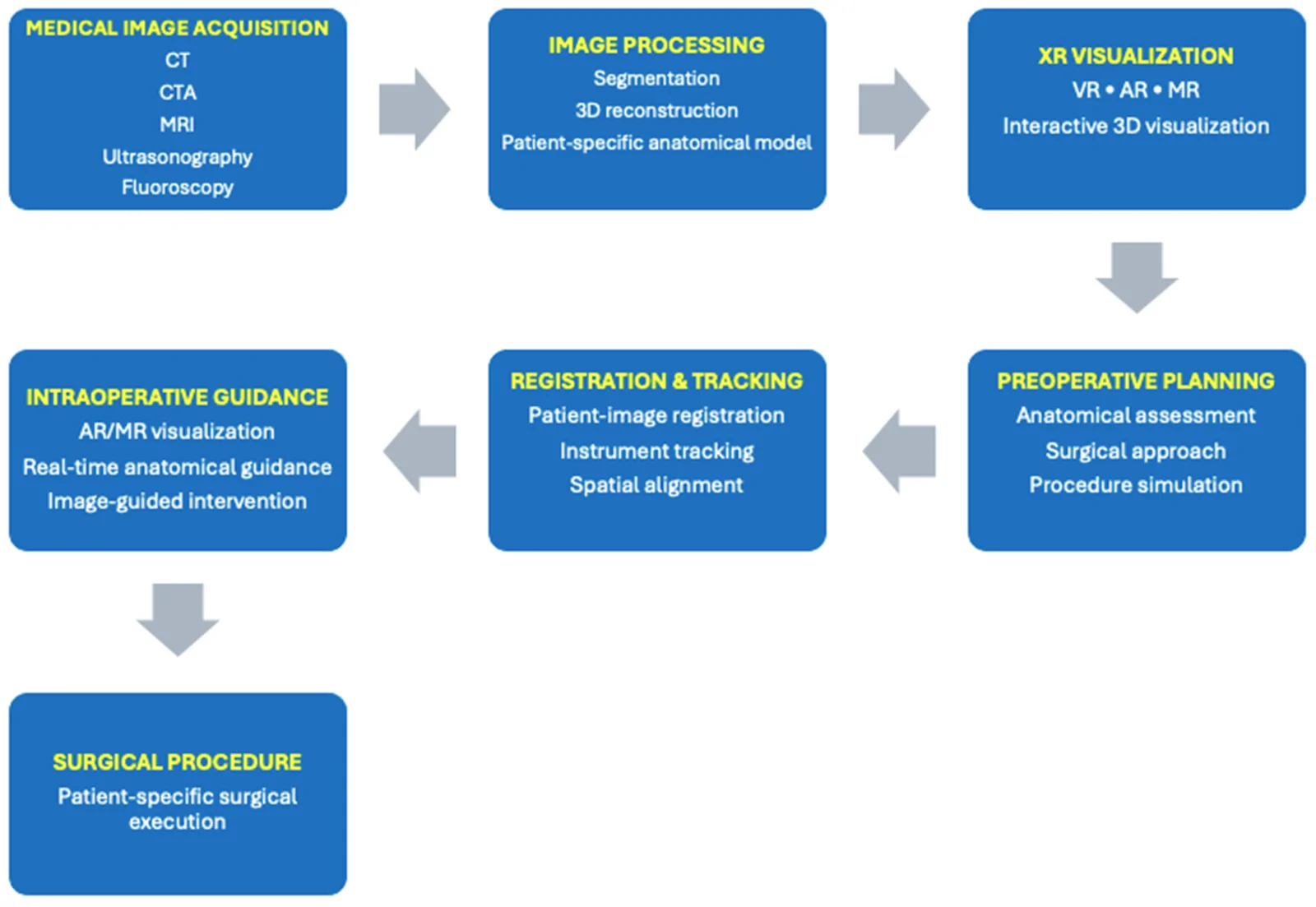
Workflow of extended reality-assisted image-guided surgery in canine patients. Schematic representation of the main stages involved in the integration of extended reality (XR) technologies into the surgical workflow, from medical image acquisition and patient-specific three-dimensional reconstruction to XR visualization, preoperative planning, image-to-patient registration and tracking, intraoperative guidance, and surgical execution. CT, computed tomography; CTA, computed tomography angiography; MRI, magnetic resonance imaging; ultrasonography; VR, virtual reality; AR, augmented reality; MR, mixed reality.

The workflow of XR-assisted image-guided surgery, from medical image acquisition and three-dimensional reconstruction to XR visualization, preoperative planning, image-to-patient registration and tracking, intraoperative guidance, and surgical execution, is summarized in Figure 2 for canine applications.

#### 3.1.2. Extended Reality for Intraoperative Navigation and Surgical Guidance

While preoperative planning currently represents the most clinically developed applications of XR in canine surgery, the integration of XR technologies into the intraoperative environment remains at an early stage of development. Intraoperative XR aims to provide surgeons with real-time visualization of patient-specific anatomical information by superimposing virtual structures onto the operative field or by displaying three-dimensional anatomical models synchronized with the patient’s position. These systems seek to overcome one of the principal limitations of conventional image-guided surgery, namely the need for surgeons to repeatedly shift their attention between the surgical field and external imaging displays.

One of the first veterinary studies specifically investigating intraoperative AR was recently published by Tipirneni et al., who evaluated the use of AR smart glasses for spatial tracking and intraoperative annotation during surgical procedures. The system allowed virtual annotations to be projected directly within the surgeon’s field of view while simultaneously tracking the position of surgical instruments. The authors demonstrated that direct AR-assisted visualization was not only technically feasible but significantly improved precision, yielding a lower mean distance error compared to traditional screen transfer and increasing accurate area coverage without compromising procedural speed. Although the study primarily focused on technical validation rather than clinical outcome assessment, it highlighted the potential of wearable AR devices to enhance intraoperative guidance in veterinary surgery [27].

To date, intraoperative AR applications in small animal surgery have focused on both rigid maxillofacial structures and soft-tissue interventions. Zhou et al. pioneered an in vivo craniomaxillofacial application by evaluating an AR-assisted robotic system for mandibular osteotomy. Four novice surgeons performed 20 bilateral drillings on two live dogs using a stereoscopic head-mounted display (nVisor ST60) and a 7-degree-of-freedom robotic arm. To protect the inferior alveolar nerve, a haptic force-feedback algorithm automatically halted drilling upon bone penetration. Postoperative analysis demonstrated high technical accuracy, with mean entrance and target errors of 1.04 ± 0.19 mm and 1.22 ± 0.24 mm, respectively. However, limitations included a small sample size, potential drilling-induced thermal damage, and a rigid human–computer interface [28]. Similarly, Li et al. investigated an AR navigation system for pulmonary nodule localization using a canine experimental model. Patient-specific CT images were used to generate virtual 3D reconstructions that were accurately registered with the surgical field through an AR navigation platform. The system enabled precise localization of pulmonary targets before thoracoscopic surgery, reducing localization errors while demonstrating excellent spatial correspondence between virtual and real anatomical structures. Although performed in an experimental setting, this work represents one of the most advanced examples of AR-assisted surgical navigation currently available in veterinary-related research [29].

George et al. developed an MR fluorescence-guided surgical platform integrating a bioinspired multispectral imaging sensor with a head-mounted display. Following validation on phantoms and experimental cancer models, the system was evaluated during surgical treatment of canine head and neck tumors. Fluorescent information was projected directly into the surgeon’s field of view as mixed-reality overlays, improving visualization of fluorescent targets while integrating seamlessly into the surgical workflow. This study represents one of the first peer-reviewed demonstrations of MR-assisted image-guided oncologic surgery in veterinary patients [30].

#### 3.1.3. Simulation and Training as a Bridge to Clinical Translation

Although the primary focus of this review is image-guided surgery, XR-based education and procedural training are included because they currently represent the most extensively investigated application of XR technologies in veterinary medicine. Educational applications of VR, AR, and MR have been more extensively explored for surgical simulation, procedural rehearsal, anatomical orientation, and image-guided skill acquisition [20,21].

Immersive VR environments enable repeated practice of laparoscopic and endoscopic procedures in a controlled setting without compromising animal welfare. Several studies evaluating laparoscopic ovariectomy simulators and basic laparoscopic skills have reported improvements in hand–eye coordination, instrument manipulation, procedural efficiency, and operator confidence among veterinary students and trainees [31,32,33,34,35,36,37]. Interestingly, a direct technological comparison by Fransson et al. investigated the construct and concurrent validity of instrument motion metrics in veterinary trainees, demonstrating that AR simulators were superior to VR systems in assessing complex skills like path length and economy of motion, whereas the VR system was validated primarily for time scores [38].

Beyond minimally invasive surgery, XR technologies have also been applied to image-guided procedural training. AR and MR platforms have been explored for image-guided procedural training, including ultrasound-guided regional anesthesia, ultrasonography, and diagnostic imaging interpretation by overlaying relevant anatomical information onto physical simulators or real-world training environments. These systems can enhance spatial orientation, facilitate needle trajectory planning, and improve the correlation between imaging findings and underlying anatomy, thereby supporting the acquisition of image-guided procedural skills [20,31,39,40].

XR-based simulation also offers important advantages within the framework of competency-based veterinary education [41]. Many contemporary XR platforms provide objective performance metrics, immediate feedback, and repeated opportunities for procedural repetition, while contributing to the implementation of the 3Rs (Replacement, Reduction, and Refinement), specifically Replacement, by reducing the need for live animals during preclinical training. These features are particularly relevant for competency-based surgical curricula, where standardized assessment and repeated deliberate practice are fundamental components of skill acquisition. However, most veterinary XR education studies are still limited by small sample sizes, heterogeneous study designs, and outcome measures based primarily on user satisfaction or simulator performance [42]. Objective assessments of long-term knowledge retention, transfer of skills to clinical surgery, and educational cost-effectiveness remain scarce [41,43]. In this context, Hunt et al. conducted a randomized controlled trial involving 44 veterinary students to evaluate whether integrating a stereoscopic VR application into a traditional surgical curriculum improved performance during their first canine ovariohysterectomy. Interestingly, the study found no significant differences in surgical performance scores or operative times between the VR and control groups, highlighting that simply adding immersive tools to an already comprehensive curriculum may not automatically translate into superior hands-on clinical skills [44]. 

Although these educational applications are primarily educational rather than clinical, they reproduce the same digital workflow used in XR-assisted surgery, including image segmentation, 3D reconstruction, spatial registration, and immersive visualization, thereby facilitating the acquisition of technical and cognitive skills that are directly transferable to image-guided surgical procedures. For these reasons, surgical education can represent an important gateway for the clinical implementation of XR technologies in veterinary medicine, providing the technological framework and user familiarity necessary for their future integration into image-guided surgical practice.

#### 3.1.4. Current Clinical Evidence

Despite the increasing interest in XR technologies, the body of clinical evidence available in small animal surgery is still remarkably limited [24,25,26]. Furthermore, most studies have primarily focused on technical feasibility rather than patient-centered clinical outcomes [26].

The available studies encompass a range of applications, including patient-specific anatomical visualization, preoperative planning, intraoperative image guidance, and procedure-specific visualization [24,25,26,28,29,30]. The reported applications involve complex surgical scenarios such as congenital vascular anomalies, thoracic procedures, surgical oncology, and other image-guided interventions [24,26].

Overall, the clinical literature remains heterogeneous in terms of study design, XR technology, imaging modality, and surgical application, with evidence ranging from experimental models and technical demonstrations to individual clinical cases [24,25,26,27,28,29,30]. Taken together, these studies indicate that XR has been applied across different stages of the surgical workflow, from anatomical assessment and procedural planning to intraoperative visualization. However, the available literature remains predominantly exploratory, and the clinical applications reported to date are still relatively few [24,25,26].

## 4. Discussion

The present review shows that XR technologies have entered the field of veterinary surgery, although their clinical implementation remains at an early stage. While the number of publications has steadily increased over the last decade, the available literature is characterized by a predominance of proof-of-concept studies, technical feasibility reports, and isolated clinical cases rather than prospective clinical investigations [20,21]. Consequently, current evidence supports the technical feasibility of XR-assisted veterinary surgery but is still insufficient to establish its routine clinical value.

An important finding emerging from the present review is the imbalance between educational and clinical applications. Most veterinary publications focus on simulation, anatomical visualization, and procedural training, whereas only a limited number describe the direct use of XR during surgical planning or intraoperative image-guided procedures. This distribution probably reflects the natural evolution of emerging surgical technologies, where educational implementation generally precedes widespread clinical adoption. The current evolution of XR in veterinary surgery follows a progressive pathway, with educational simulation representing the most extensively investigated application, followed by preoperative planning and, more recently, early clinical implementation of image-guided surgical procedures [20,24,26,27]. The studies by Stoner et al. [24] and Saunders et al. [25] support the potential value of interactive three-dimensional visualization for the assessment and planning of complex cardiovascular procedures, whereas the clinical case reported by Cupido et al. [26] represents a further step toward the integration of XR into an actual surgical workflow. These studies suggest that the most immediate clinical benefit of XR can lie in improving the interpretation of patient-specific anatomy and supporting preoperative decision-making rather than providing fully automated intraoperative guidance.

Nevertheless, it warrants careful consideration that the pedagogical efficacy of XR in veterinary training is highly task-dependent and not universally superior to traditional methods. For instance, while immersive platforms like MR head-mounted displays significantly enhance student satisfaction and technical proficiency in procedures requiring precise depth and angular perception, such as orthopedic screw fixation, they can offer limited benefits in delicate microsurgical tasks like corneal suturing [42]. Furthermore, a randomized controlled trial evaluating stereoscopic VR video applications failed to demonstrate a measurable improvement in objective surgical performance scores or operative times during live canine ovariohysterectomies compared to conventional training [44]. This suggests that simply introducing immersive visual tools into an already rigorous curriculum does not automatically translate into enhanced tactile proficiency or superior hands-on clinical skills. Consequently, future veterinary educational frameworks should focus on integrating high-fidelity simulators that combine visual immersion with advanced haptic feedback, ensuring that XR implementation addresses specific cognitive or spatial training deficits rather than serving as a generic replacement for traditional instructional modalities. The findings of Fransson et al. [38] further support this task-dependent interpretation, as differences in the validity of instrument-motion metrics between VR and AR systems indicate that the choice of XR platform can influence the type of skills that can be effectively assessed. Therefore, the educational evidence should be considered as supportive of XR as a complementary training modality rather than evidence of its universal superiority.

One of the most striking observations emerging from this review is the considerable gap between human and veterinary medicine regarding the clinical implementation of XR technologies. Several factors likely contribute to this discrepancy [13,14,15,16,17,18,19]. The veterinary healthcare market is considerably smaller, limiting commercial investment in dedicated XR technologies. Furthermore, the relatively low number of highly complex surgical procedures performed within individual veterinary centers makes prospective multicenter clinical trials difficult to conduct. Finally, successful development of XR systems requires close collaboration among veterinary surgeons, radiologists, biomedical engineers, and computer scientists, multidisciplinary teams that could be relatively uncommon in veterinary institutions. The studies currently available also suggest that veterinary applications frequently rely on technologies initially developed for human medicine, which can further limit the availability of systems specifically optimized for the anatomical variability, workflow and economic characteristics of veterinary practice [20]. This may partly explain why several veterinary studies remain at the proof-of-concept or experimental stage despite the more advanced clinical implementation observed in human surgery.

Beyond its direct surgical applications, immersive visualization can also enhance communication with pet owners by facilitating the explanation of complex anatomy, planned surgical procedures, and expected outcomes through patient-specific 3D models. Although this potential has been primarily described in human medicine, similar benefits can be anticipated in veterinary practice, particularly during preoperative counselling and the informed consent process [13]. In this contest, XR could provide an additional communication tool by allowing owners to visualize patient-specific anatomical abnormalities and proposed surgical approaches.

Despite encouraging technological developments, several important barriers continue to hinder widespread clinical implementation of XR. Accurate image registration is one of the principal technical limitations, particularly during soft tissue surgery where respiratory motion, tissue deformation, and surgical manipulation continuously modify anatomical relationships [20]. In addition, maintaining accurate spatial correspondence between virtual models and the operative field remains challenging during dynamic surgical procedures, highlighting the need for more robust tracking algorithms and real-time registration techniques before routine clinical adoption [20]. Generation of patient-specific 3D models continues to be a time-consuming process because image segmentation, registration, and model refinement often require substantial manual input from experienced operators before surgical visualization can be achieved [13]. In addition, the high costs associated with advanced imaging equipment, dedicated software, and head-mounted displays currently limit the implementation of XR technologies primarily to referral hospitals, academic institutions, and specialized research centers [20]. Finally, the quality of the available evidence remains relatively low. Most veterinary studies evaluate technical feasibility rather than clinically meaningful outcomes such as operative time, surgical precision, complication rates, or long-term patient outcome. Consequently, prospective comparative clinical studies remain one of the major priorities for future veterinary XR research [21]. The experimental studies by Zhou et al. [28] and Li et al. [29] illustrate this distinction particularly well: both demonstrated promising levels of spatial accuracy and successful registration in controlled experimental settings, but neither study can establish whether these technical improvements translate into clinically relevant benefits in routine veterinary patients. Similarly, the study by George et al. [30] demonstrates the feasibility of integrating MR with fluorescence-guided surgery, but further investigation is required to determine whether this additional visualization improves oncological or surgical outcomes. Thus, technical accuracy should be considered an important prerequisite for clinical translation, but not a surrogate for clinical effectiveness.

The findings of the present review clearly identify several priorities for future investigation. Prospective multicenter clinical trials should evaluate objective surgical outcomes, including operative time, procedural accuracy, postoperative complications, and learning curves. Standardized reporting guidelines would facilitate comparison among studies while improving the overall quality of evidence [21]. Future technological developments are expected to focus on improving image registration accuracy, reducing workflow complexity, enhancing real-time visualization, and facilitating integration between XR platforms and surgical navigation systems. Advances in artificial intelligence can further automate image segmentation and registration, potentially improving the clinical feasibility of XR-assisted procedures [13,20]. Finally, stronger collaboration between veterinary surgeons, biomedical engineers, imaging specialists, and computer scientists will be essential for translating promising technological innovations into clinically validated surgical tools. Such multidisciplinary collaboration has been recognized as a key prerequisite for the successful development and clinical implementation of XR technologies in surgery [13].

Several limitations should be considered when interpreting the findings of this review. First, the available veterinary literature is limited and heterogeneous, encompassing technical feasibility studies, experimental models, educational investigations, pilot studies, and individual clinical reports. The XR technologies, imaging modalities, surgical applications, and outcome measures also vary substantially among studies, precluding quantitative synthesis or direct comparison of results. In addition, the present work was designed as a narrative review and therefore did not include a formal systematic screening protocol or standardized risk-of-bias assessment. Finally, prospective comparative clinical studies evaluating clinically relevant outcomes in canine patients remain scarce. These limitations highlight the need for standardized methodologies and prospective clinical investigations before the routine clinical value of XR-assisted surgery can be established.

## 5. Conclusions

Extended reality is emerging as a potentially valuable technology in canine image-guided surgery, enabling the conversion of conventional two-dimensional imaging into immersive, patient-specific 3D environments that improve anatomical understanding and support surgical decision-making. Current evidence demonstrates promising applications in preoperative planning, intraoperative guidance, image-guided interventions, and surgical education. However, clinical adoption remains limited, with most available studies consisting of technical reports, pilot investigations, or isolated case reports.

The widespread implementation of XR in canine surgery will require robust prospective clinical studies, standardized workflows, and technological advances that improve image registration, workflow efficiency, and system interoperability. As these challenges are progressively addressed, XR has the potential to become an integral component of precision canine surgery, supporting more accurate planning, safer procedures, and improved patient-specific surgical care.

## Data Availability

No new data were created or analyzed in this study. Data sharing is not applicable to this article.

## References

[1] Privitera L., Paraboschi I., Dixit D., Arthurs O.J., Giuliani S. (2021). Image-guided surgery and novel intraoperative devices for enhanced visualisation in general and paediatric surgery: A review. Innov. Surg. Sci..

[2] Azagury D.E., Dua M.M., Barrese J.C., Henderson J.M., Buchs N.C., Ris F., Cloyd J.M., Martinie J.B., Razzaque S., Nicolau S. (2015). Image-guided surgery. Curr. Probl. Surg..

[3] Vignoli M., Saunders J.H. (2011). Image-guided interventional procedures in the dog and cat. Vet. J..

[4] Avner A., Herrtage M.E., Segal U. (2019). Ultrasound guided trans-hepatic coil embolization of an intrahepatic portosystemic shunt in a dog. Isr. J. Vet. Med..

[5] Caivano D., Birettoni F., Marchesi M.C., Moretti G., Corda A., Petrescu V.F., Porciello F., Bufalari A. (2019). Septic pericarditis and cardiac tamponade caused by migrating intrathoracic grass awn in an English setter dog. Isr. J. Vet. Med..

[6] Valderrama V.F.R., Valeri F., Caivano D., Moretti G., Bufalari A. (2026). Ultrasound-guided minimally invasive thoracotomy for removal of migrating grass Awn in a Cat. Vet. Res. Commun..

[7] Bufalari A., Moretti G., Monti E., Garofanini L., Angeli G., Porciello F., Marchesi M.C., Caivano D. (2022). Intracardiac Porcupine Quill Migration in a Dog: Multimodality Imaging Findings and Surgical Management. Vet. Sci..

[8] Carroll J., Coutermarsh-Ott S., Klahn S.L., Tuohy J., Barry S.L., Allen I.C., Hay A.N., Ruth J., Dervisis N. (2022). High intensity focused ultrasound for the treatment of solid tumors: A pilot study in canine cancer patients. Int. J. Hyperth..

[9] Ruger L., Yang E., Coutermarsh-Ott S., Nightengale M., Hsueh A., Vickers E.R., Ciepluch B., Vlaisavljevich E., Dervisis N., Klahn S. (2026). Pilot Study of Partial Tumor Ablation Using Thermal High-Intensity Focused Ultrasound (HIFU) in Feline Soft Tissue Sarcomas. Animals.

[10] Griffin M.A., Culp W.T.N., Buote N.J. (2025). The role of minimally invasive surgery in oncology—Part 1: Laparoscopy. Vet. Oncol..

[11] Griffin M.A., Culp W.T.N., Buote N.J. (2025). The role of minimally invasive surgery in oncology—Part 2: Thoracoscopy. Vet. Oncol..

[12] Sasaki K., Medan M.S., Azuma T., Kawabata K., Shimoda M., Umemura S. (2006). Effect of echo-guided high-intensity focused ultrasound ablation on localized experimental tumors. J. Vet. Med. Sci..

[13] Zhang J., Lu V., Khanduja V. (2023). The impact of extended reality on surgery: A scoping review. Int. Orthop..

[14] Morimoto T., Kobayashi T., Hirata H., Otani K., Sugimoto M., Tsukamoto M., Yoshihara T., Ueno M., Mawatari M. (2022). XR (Extended Reality: Virtual Reality, Augmented Reality, Mixed Reality) Technology in Spine Medicine: Status Quo and Quo Vadis. J. Clin. Med..

[15] Khubzan W.D., Alharati S.A., Aljuaid R.W., Algethami D.S., Alotibi S.S., Alotaibi H.M., Aljuaid S.S., Almalki R.S., Mahfouz M.E.M. (2026). The digital evolution of surgical planning: A systematic review of immersive and interactive technologies. Front. Surg..

[16] Ma L., Huang T., Wang J., Liao H. (2023). Visualization, registration and tracking techniques for augmented reality guided surgery: A review. Phys. Med. Biol..

[17] Murphy E., Cahill R.A. (2025). Technological advances in intra-operative navigation: Integrating fluorescence, extended reality, and artificial intelligence. J. Clin. Med..

[18] Nowak R., Nowak M., Rękas M., Ali M.J. (2025). Intraoperative use of mixed reality (MR) in humans across surgical disciplines: A major review. 3D Print. Med..

[19] Li Y., Li M., Zheng S., Yang L., Peng L., Fu C., Chen Y., Wang C., Chen C., Li B. (2024). Exploring the dynamics of user experience and interaction in XR-enhanced robotic surgery: A systematic review. Front. Virtual Real..

[20] Aghapour M., Bockstahler B. (2022). State of the art and future prospects of virtual and augmented reality in veterinary medicine: A systematic review. Animals.

[21] Daungsupawong H., Wiwanitkit V. (2026). Virtual reality: Applications in veterinary science. Vet. Med. Sci..

[22] Green B.N., Johnson C.D., Adams A. (2006). Writing narrative literature reviews for peer-reviewed journals: Secrets of the trade. J. Chiropr. Med..

[23] Klasen J.R.S., Thatcher G.P., Bleedorn J.A., Soukup J.W. (2022). Virtual surgical planning and 3D printing: Methodology and applications in veterinary oromaxillofacial surgery. Front. Vet. Sci..

[24] Stoner C.H., Wesselowski S., Gordon S.G., Saunders A.B. (2025). Application of an interactive, stereoscopic three-dimensional viewer to evaluate atrial septal defects in five dogs. J. Vet. Cardiol..

[25] Saunders A.B., Doocy K.R., Birch S.A. (2019). A pictorial view of the three-dimensional representation and comparative two-dimensional image orientation derived from computed tomography angiography in a dog with a patent ductus arteriosus. J. Vet. Cardiol..

[26] Cupido S., Valeri F., Nicoli S., Bargellini P., Caivano D., Birettoni F., Bortolotti A., Rishniw M., Porciello F. (2025). A new technology for a novel clinical approach in a dog with a complex vascular anomaly: The “extended reality”. Vet. Res. Commun..

[27] Tipirneni Y., Arzi B., Blandino A., Skouritakis C.T., Goldschmidt S. (2026). Augmented reality glasses improve real-time spatial tracking and surface-level annotations on the canine head. Am. J. Vet. Res..

[28] Zhou C., Zhu M., Shi Y., Lin L., Chai G., Zhang Y., Xie L. (2017). Robot-assisted surgery for mandibular angle split osteotomy using augmented reality: Preliminary results on clinical animal experiment. Aesthetic Plast. Surg..

[29] Li C., Zheng Y., Yuan Y., Li H. (2021). Augmented reality navigation-guided pulmonary nodule localization in a canine model. Transl. Lung Cancer Res..

[30] George M.B., Lew B., Liang Z., Blair S., Zhu Z., Cui N., Ludwig J., Zayed M., Selmic L., Gruev V. (2023). Fluorescence-guided surgical system using holographic display: From phantom studies to canine patients. J. Biomed. Opt..

[31] Hottmann N.M., Fransson B.A. (2025). Effects of short-course virtual reality or video-box training on basic laparoscopic skills and simulated surgical performance in veterinary students. Vet. Surg..

[32] Fransson B.A., Ragle C.A. (2010). Assessment of laparoscopic skills before and after simulation training with a canine abdominal model. J. Am. Vet. Med. Assoc..

[33] Fransson B.A., Ragle C.A., Bryan M.E. (2010). A laparoscopic surgical skills assessment tool for veterinarians. J. Vet. Med. Educ..

[34] Guaraná J.B., Aytaç G., Müller A.F., Thompson J., Freitas S.H., Lee U.Y., Lozanoff S., Ferrante B. (2023). Extended reality veterinary medicine case studies for diagnostic veterinary imaging instruction: Assessing student perceptions and examination performance. Anat. Histol. Embryol..

[35] Micșa C., Vițălaru B.A., Dragomirișteanu I., Bîrțoiu I.A. (2023). Different training methods used to improve laparoscopic skills in veterinary medicine. Alveus.

[36] Danner A.L., Song M.K., Fracka A.B., Dickson R., Fransson B. (2026). Validity assessment for veterinary use of human laparoscopic cholecystectomy training models. Am. J. Vet. Res..

[37] Redander J., Enlund K., Höglund O.V. (2025). The development of a novel virtual reality simulation module for canine laparoscopic ovariectomy. Acta Vet. Scand..

[38] Fransson B.A., Ragle C.A. (2016). Instrument motion metrics for laparoscopic skills assessment in virtual reality and augmented reality simulators. Vet. Surg..

[39] Hung C.Y., Chang K.V., Ozcakar L., Hsiao M.Y., Wang T.G., Chen W.S. (2011). Ultrasound-guided injection techniques for the low back and hip joint. Chang Gung Med. J..

[40] McGaffey M., Shafford H.L., Meadows L., Fox S., Boettcher L.M., Hostutler R.A. (2024). Assessment of an ultrasound skill simulator in teaching ultrasound-guided paracentesis: A prospective observational study. J. Vet. Med. Educ..

[41] Zhang J., Lauinger A., Shrivastava R., Falak A., Abraskin K., Burnette P., Galvez R. (2026). Use of extended reality head-mounted displays in healthcare education: A scoping review. Front. Med..

[42] Sasaki N., Lee S. (2025). Educational dissemination in the digital age through the TranspaVet project: Evaluation of students’ satisfaction and instructional efficacy. J. Vet. Med. Educ..

[43] Oviedo-Peñata C.A., Tapia-Araya V., Lemos Z., Riaño-Benavides C.E., Case J.B., Maldonado-Estrada J.G. (2020). Validation of training and acquisition of surgical skills in minimally invasive surgery in small animal surgery: A comprehensive review. Front. Vet. Sci..

[44] Hunt J.A., Heydenburg M., Anderson S.L., Thompson R.R. (2020). Does virtual reality training improve veterinary students’ first canine surgical performance?. Vet. Rec..

